# Improvement of Sepsis by Hepatocyte Growth Factor, an Anti-Inflammatory Regulator: Emerging Insights and Therapeutic Potential

**DOI:** 10.1155/2012/909350

**Published:** 2012-02-28

**Authors:** Shinya Mizuno, Toshikazu Nakamura

**Affiliations:** ^1^Division of Virology, Department of Microbiology and Immunology, Osaka University Graduate School of Medicine, 2-2-B7 Yamadaoka, Suita 565-0871, Japan; ^2^Kringle Pharma Joint Research Division for Regenerative Drug Discovery, Center for Advanced Science and Innovation, Osaka University, 2-1 Yamadaoka, Suita 565-0871, Japan

## Abstract

Sepsis-induced multiple organ failure (MOF) is the most frequent lethal disease in intensive care units. Thus, it is important to elucidate the self-defensive mechanisms of sepsis-induced MOF. Hepatocyte growth factor (HGF) is now recognized as an organotrophic factor, which is essential for organogenesis during embryonic growth and regeneration in adulthood. HGF production is enhanced in response to infectious challenges, but the increase in endogenous HGF levels is transient and insufficient, with a time lag between tissue injuries and HGF upregulation, during progression of septic MOF. Thus, administration of active-formed HGF might be a new candidate for therapeutic development of MOF. HGF has an ability to target endotoxin-challenged macrophages and inhibits the upregulation of inflammatory cytokines through nuclear factor-*κ*B-inactivated mechanisms. HGF also targets the endothelium and epithelium of various organs to suppress local inflammation, coagulation, and apoptotic death. This paper summarizes the novel mechanisms of HGF for attenuating sepsis-related pathological conditions with a focus on sepsis-induced MOF.

## 1. Introduction

In the first 10 years of the 21st century, the prevalence of sepsis increased worldwide due to an emergence of infectious disorders. Multiple organ failure (MOF) often occurs and progresses during septic pathological conditions such as hypercoagulation and inflammation [1]. In particular, systemic inflammatory response syndrome (SIRS) is a clinical feature of a generalized inflammatory reaction in organs that are distant from the initiating insult [2]. Approximately, 700,000 hospitalized patients develop sepsis each year in the United States, resulting in more than 210,000 deaths; this number accounts for 10% of all deaths annually and exceeds the number of deaths due to myocardial infarction [3]. Importantly, acute renal failure (ARF) and acute respiratory distress syndrome (ARDS) play a significant role in the setting of MOF during severe sepsis [3, 4]. Moreover, hepatic injury sometimes occurs as a consequence of continuous infection or shock, systemic and local inflammation, and hypoxia [5].

From the viewpoint of pathogenesis, SIRS is inducible by immune cells, especially by activated macrophages, via an innate immune mechanism. For example, lipopolysaccharide (LPS) is localized in the outer membranes of Gram-negative bacteria and acts as endotoxins to elicit strong immune responses in the host via binding to Toll-like receptor 4 (TLR4) that is expressed mainly on macrophages [6]. The binding of LPS to its receptor, TLR4, provokes transductions of intracellular signaling leading to an activation of nuclear factor (NF)-*κ*B, a key transcriptional factor for triggering inflammation, and then proinflammatory cytokines, such as tumor necrosis factor-*α* (TNF-*α*) and interleukin (IL-)1, 6, and 18, are released from macrophages to blood. Under such a systemic inflammatory condition, MOF-related lethal events become evident not only in macrophages (i.e., an initial step for cytokine storm) but also in vessels (i.e., an early step for edema and thrombosis) and organ tissues (i.e., an advanced step for apoptosis, necrosis, and loss of function). These pathological circuits are required to establish MOF-related conditions [3–5]. Thus, several approaches to intercept the MOF-related circuits may be useful for the improvement in septic mortality.

Hepatocyte growth factor (HGF) was originally identified and cloned as a potent mitogen for hepatocytes [7–9]. HGF acts on various types of cells through its receptor, c-Met, and exhibits pleiotropic activities during embryogenesis and tissue repair [10–15]. For instance, HGF protects the parenchyma by stimulation of hepatocyte proliferation and inhibition of functional cell loss during liver injury [14, 15]. Indeed, endogenous and exogenous HGF prevents acute hepatic failure in rodents, associated with the anti-apoptotic and anti-necrotic effect of HGF on hepatocytes [10, 15]. During sepsis, plasma HGF levels gradually increase in septic patients [16], while macrophages acquire high levels of c-Met *in vitro* and *in vivo* (animal models) [17–20]. Recent reports delineated the novel anti-inflammatory effects of HGF on various types of cells, including macrophages. Of interest, LPS challenge increased not only TLR4 expression but also HGF and c-Met production both *in vitro* [17, 18] and *in vivo* (animal models) [19, 20], thereby suggesting a possible physiological effect of HGF on LPS-TLR4 signaling. The present paper focuses on both the emerging roles of HGF in sepsis and the therapeutic potential of HGF-c-Met signaling to prevent or reverse MOF-related pathological conditions.

## 2. Biological Activity of HGF through Its Receptor, c-Met

**Figure 1:**
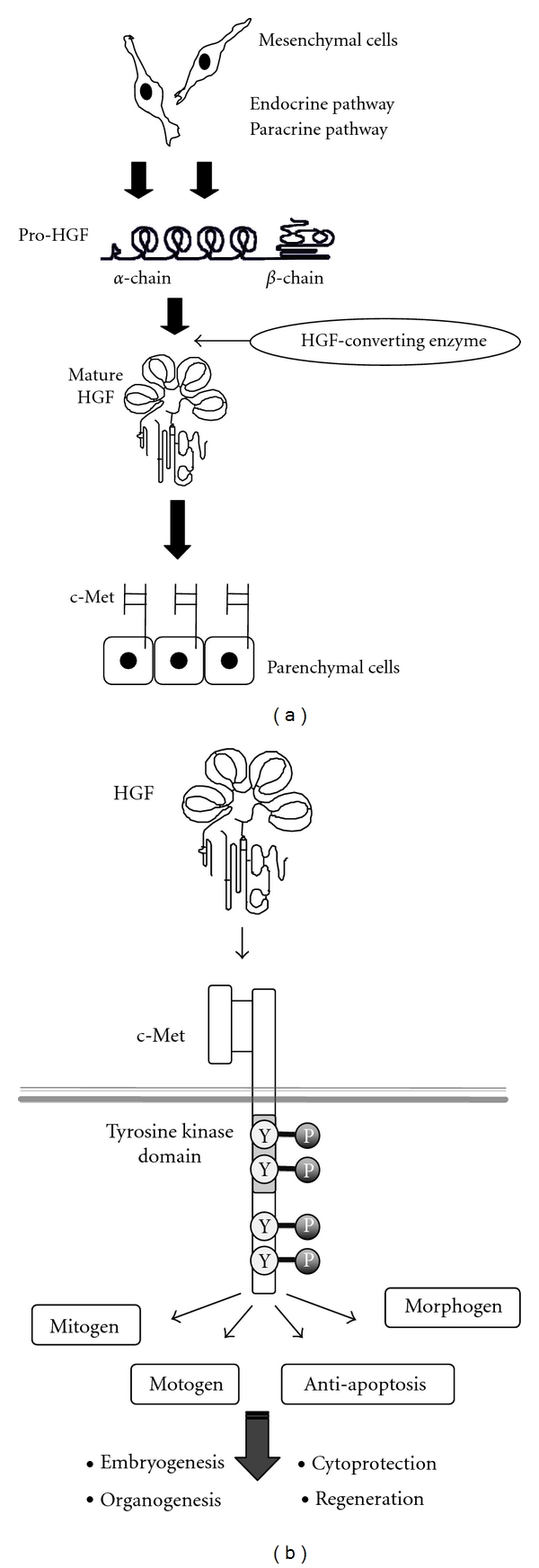
Production and multifaceted functions of HGF. (a) Production, activation, and delivery of HGF. HGF is produced in mesenchymal cells such as fibroblasts. HGF is secreted in an inactive form (i.e., pro-HGF). Under injurious conditions, pro-HGF is converted to mature HGF by HGF-converting enzymes, such as uPA. HGF is delivered to injured sites via endocrine and paracrine pathways [10]. (b) Multifaceted biological actions of HGF, mediated via c-Met tyrosine phosphorylation, are outlined. Induction of mitogenic, motogenic, and morphogenic (3 M) effects of HGF are essential for organ development and regeneration. Anti-apoptotic effects are required for protection against various types of organ injuries.

In the mid- to late-1980s, HGF was identified and cloned as a mitogen in the primary culture of rat hepatocytes [7–9]. On the other hand, c-Met, a proto-oncogene product, was identified as a high affinity receptor specific for HGF [21, 22]. Binding of HGF to c-Met induces activation of tyrosine kinase, which results in biological activities on a wide variety of cells, including mitogenic, motogenic, and morphogenic activities (Figure 1). In addition to these regenerative effects, anti-apoptotic and anti-inflammatory roles of HGF have been widely demonstrated *in vitro* and *in vivo*, as described below.

### 2.1. Anti-Apoptotic Effects

Apoptosis is an important event in the loss of parenchymal components, and Fas signaling is involved in this process. Of note, HGF inhibits Fas-mediated apoptosis [23, 24]. Treatment of primary cultured hepatocytes with a specific Fas agonist (Jo-2) rapidly induced apoptosis via a caspase-3-activated pathway. In contrast, HGF strongly inhibited the caspase-3 activation induced by Jo-2 in mouse hepatocytes via the induction of anti-apoptotic molecules such as Bcl-xL [23]. Likewise, HGF induces the expression of myeloid cell leukemia-1 (Mcl-1), which is another member of the Bcl-2 family proteins in hepatocytes [25]. In addition, FLICE-like inhibitory protein (FLIP) negatively regulates the Fas signaling pathway by interfering with activation of caspase-8. Upon activation of Fas, FLIP is rapidly degraded via the proteasome pathway, whereas HGF prevents degradation of FLIP in a PI3K/Akt-dependent pathway [26]. Such anti-apoptotic effects of HGF contribute to organ protection, especially after the onset of cytokine storm, as discussed later (see Section 4).

### 2.2. Anti-Inflammatory Functions

One of the most key results related to novel functions of HGF during sepsis is that HGF directly targets immune cells, including macrophages and lymphocytes that highly express HGF-receptor, c-Met, under inflammatory conditions. Such direct anti-inflammatory effects of HGF are considered to be the key mechanism whereby HGF inhibits cytokine storm in an initial stage of sepsis. In this section, we summarize biological activities of HGF, which are required for inhibiting the progression of SIRS-related pathological conditions.

**Macrophages**

There is now ample evidence that macrophages acquire c-Met after LPS challenge *in vitro* and *in vivo* [17–20]. Furthermore, sepsis-mediated thrombosis and hypoxia also enhance c-Met expression by macrophages through upregulating hypoxia-inducible factor-1 (HIF-1), a transcription factor for transcription of c-Met mRNA [27]. HGF produces several effects on macrophages. The most highlighted finding is that HGF inhibits LPS-mediated production of proinflammatory cytokines, such as TNF-*α*, IL-1*β*, IL-6, and IL-18 [28, 29]. Moreover, HGF induces macrophage differentiation with an anti-inflammatory phenotype (type-M2), rather than with a proinflammatory phenotype (type-M1) [30]. These effects lead to a minimization of MOF, as discussed later (see Sections 4 and 5).

**Endothelium**

Under septic conditions, neutrophils play a key role for tissue destruction via release of apoptotic enzymes, such as elastase. During the process of neutrophil infiltration, vascular endothelial cells provide an important anchorage (such as ICAM-1 and E-selectin) for neutrophil adhesion on endothelial cell surfaces, resulting in rolling and extravasation [31]. Using a mouse model of renal ischemia, we first provided evidence that HGF suppresses the neutrophil extravasation via inhibiting TNF-*α*-induced ICAM-1 expression on endothelium (e.g., HUVEC model) [32]. These results were also reproducible in other models, where HGF counteracted the endothelial E-selectin expression and this effect attenuated chronic kidney injury in rodents [33, 34]. Thus, HGF-mediated inhibition of neutrophil-adherent molecules (such as ICAM-1/E-selectin) is contributable for reducing neutrophil infiltration and subsequent attack under inflammatory states, especially at an early phase of MOF, as discussed later.

### 2.3. Anti-Coagulant Properties

In addition to inflammatory phenomena, disseminated intravascular coagulation (DIC) is a common event under septic conditions. Interestingly, thrombosis increases blood HGF levels by degranulation of mast cells [35], suggesting a possible physiological effect of HGF against DIC. Actually, HGF elicits anti-coagulant outcomes in several types of liver diseases [36, 37], possibly through the induction of urokinase-type plasminogen activator (uPA) that is known to stimulate fibrinolysis [38]. Furthermore, HGF is shown to inhibit edematous formation postthrombosis via Rac-dependent inhibition of the Rho pathway [39]. These effects lead to the reduction of fibrin deposition in endothelial lumens under pathological states [28, 37]. IL-6 is responsible for thrombosis during sepsis, while HGF potently inhibits IL-6 production in LPS-primed macrophages [28]. Thus, these direct and indirect effects by c-Met signaling lead to anti-coagulant outcomes in rodents with sepsis. The cells targeted by HGF and its function during inflammation and coagulation are summarized in Table 1.

## 3. Regulation and Significance of HGF Production during Sepsis

Several lines of clinical studies demonstrated that blood and local HGF levels increased in patients with infectious diseases with septic features [16, 47, 48]. Under inflammation, HGF is produced and delivered to injured tissue via systemic (i.e., endocrine) and local (i.e., paracrine) mechanisms [10, 15]. Sepsis-mediated upregulation of HGF is suggested to be an effort to minimize the manifestation of MOF, but this response may be transient, delayed and sometimes insufficient, during progression of septic MOF. Prior to discussion on the therapeutic value of HGF, we emphasize the possible role of endocrine and paracrine pathways in HGF production, because this response may lead to the elicitation of defensive responses to injury.

### 3.1. Production and Delivery of HGF during Sepsis

**(A) Endocrine System**

Under a healthy state, HGF is produced at a physiological level (0.2–0.4 ng/mL in blood) by mesenchymal cells to sustain parenchymal homeostasis, and this production is enhanced in a response to tissue injury [11]. Of interest, bacterial components, such as LPS from Gram-negative bacteria, protein A from *Staphylococcus aureus*, and fimbriae of *Porphyromonas gingivalis* stimulates HGF production in fibroblasts or macrophages through a transcriptional pathway [18, 49, 50]. Blood-born HGF can be stocked on surface of neutrophils, while LPS or TNF-*α* releases HGF from the cell surface (i.e., detachment mechanism) [51]. These transcriptional and nontranscriptional mechanisms could contribute to an endocrine delivery of HGF to injured tissues through vascular blood flow.

**(B) Paracrine System**

In addition, the paracrine system of HGF delivery production is also important as a local defensive system. Under infectious diseases, neutrophils undergo apoptosis after intake of bacteria and then are phagocytized by infiltrated macrophages. During this process, HGF transcription is enhanced in activated macrophages [52] and *de novo* synthesized HGF is delivered to neighboring epithelium via a paracrine loop. LPS is known to induce inflammatory cytokines, such as TNF-*α*, IL-1, and IL-6 via the TLR4-dependent pathway, and interestingly, these cytokines enhance HGF production in mesenchymal cells [53, 54]. Such an inflammation-dependent upregulation of HGF may also participate in paracrine (and possibly, endocrine) mechanisms for inducing self-defense responses [10, 15].

### 3.2. A Protective Role of Endogenous HGF-c-Met Axis during Sepsis

LPS stimulates the production of not only ligand HGF but also its receptor, c-Met [17]. Furthermore, LPS promotes the mitogenic effects of HGF in various organs in rats [55], suggesting that LPS also elicits compensatory responses, in contrast to the primary function (i.e., a trigger of the innate immune system). Indeed, blood HGF levels are elevated in septic mice, in association with concomitant increases of phosphorylated c-Met signals in the macrophages and renal cells [28, 29, 56]. Of note, anti-HGF IgG worsens tissue injuries in LPS-treated mice, thus suggesting that endotoxin-induced increases in HGF and c-Met are required for reducing organ destruction and dysfunction against septic stresses in animals (and possibly in humans). 

### 3.3. HGF as a Diagnostic Marker of SIRS-to-MOF

Since endogenous HGF is important for driving self-defensive system, local or systemic HGF may be useful for diagnosis of septic conditions: HGF levels significantly increase in blood or bronchoalveolar lavage fluids (BALFs) of patients during infectious diseases [11, 57]. For example, HGF is detected exclusively in BALF from patients with sepsis-related acute respiratory distress syndrome (ARDS), and the elevation in HGF levels is evident in patients with severe, rather than mild, injury [57]. Given that HGF is protective during sepsis, the increase of blood HGF may be interpreted as a compensated response, rather than a risk factor. Blood HGF is now suggested to be a sensitive marker to predict the severity of tissue injuries under clinical situations (see Table 2).

During the past 20 years, we have postulated that endogenous HGF is a key physiological regulator to minimize organ damage and dysfunction in various organs [10, 15]. In a rat model of sepsis, enhancement of HGF production was insufficient, with a time lag, after an endotoxic challenge (i.e., 3-fold increase at 12 hours after LPS treatment), associated with the onset of septic hepatitis [63]. Based on this background, we demonstrated in the late 1990s that a forced increase by supplemental therapy with HGF could prevent LPS-induced hepatitis [40]. This concept is now widely supported in rodent models of septic diseases, as follows.

## 4. Therapeutic Effects of HGF on MOF during Sepsis

Sepsis-induced MOF is the most frequent disease in the intensive care unit (ICU). In the United States, an estimated 700,000 cases of sepsis occur annually [3], resulting in 210,000 deaths; this number accounts for 10% of all deaths annually and exceeds the number of deaths due to myocardial infarction [3]. Nevertheless, there are no treatments specific to septic organ injury. We and other groups have accumulated evidence to show that HGF, an intrinsic anti-inflammatory regulator, is useful to inhibit or reverse sepsis-induced hepatitis, ARDS, and acute renal failure (ARF) at least in animal models, as described below.

### 4.1. Anti-Apoptotic and Anti-Coagulant Outcomes of HGF during Hepatitis

LPS elicits intravascular thrombosis and a local decrease in blood flow, and then parenchymal apoptosis becomes evident under such a hypoxic condition. Using LPS- and D-galactosamine (GalN-) treated mice as a “lethal” model of sepsis, we found that an early induction of anti-apoptotic molecule (such as Bcl-xL) by recombinant HGF leads to the inhibition of massive hepatocyte loss and hepatic dysfunction [40]. In this process, exogenous HGF strongly blocked mitochondrial damages and caspase-3 activation induced by LPS + GalN. As a result, 75% of septic mice were rescued by the HGF injections, while all control mice died of severe hepatic failure within 8 hours after septic challenge without HGF treatment [40]. Based on these data, we emphasized that HGF may have the potential to inhibit fulminant hepatic failure during sepsis via its potent anti-apoptotic action.

Considering that HGF stimulates fibrinolysis [38], HGF may improve thrombosis. With regard to this, Seto et al. investigated the protective effect of HGF on sinusoidal endothelial cells (SECs) in the LPS-induced liver injury of rats [44]. Indeed, fibrin was deposited mainly in SEC in the LPS-treated rats. In contrast, fibrin deposition by SEC became mild when HGF was supplemented via splenic transplantation of HGF-producing fibroblasts in endotoxemic rats [44], indicating an anti-thrombotic effect of HGF under septic conditions. Interestingly, thrombosis itself induces a rapid increase of HGF in blood through the release of mast cell granulations (including heparin) by thrombin stimulation [35]. Such a circuit between thrombosis and HGF upproduction is thought to be a counteractive response to arrest the progression of thrombosis. Thus, early administration of recombinant HGF may be one of new candidates for therapeutic development of DIC during septic conditions.

### 4.2. Prevention of ARDS by HGF Treatment

**Figure 2:**
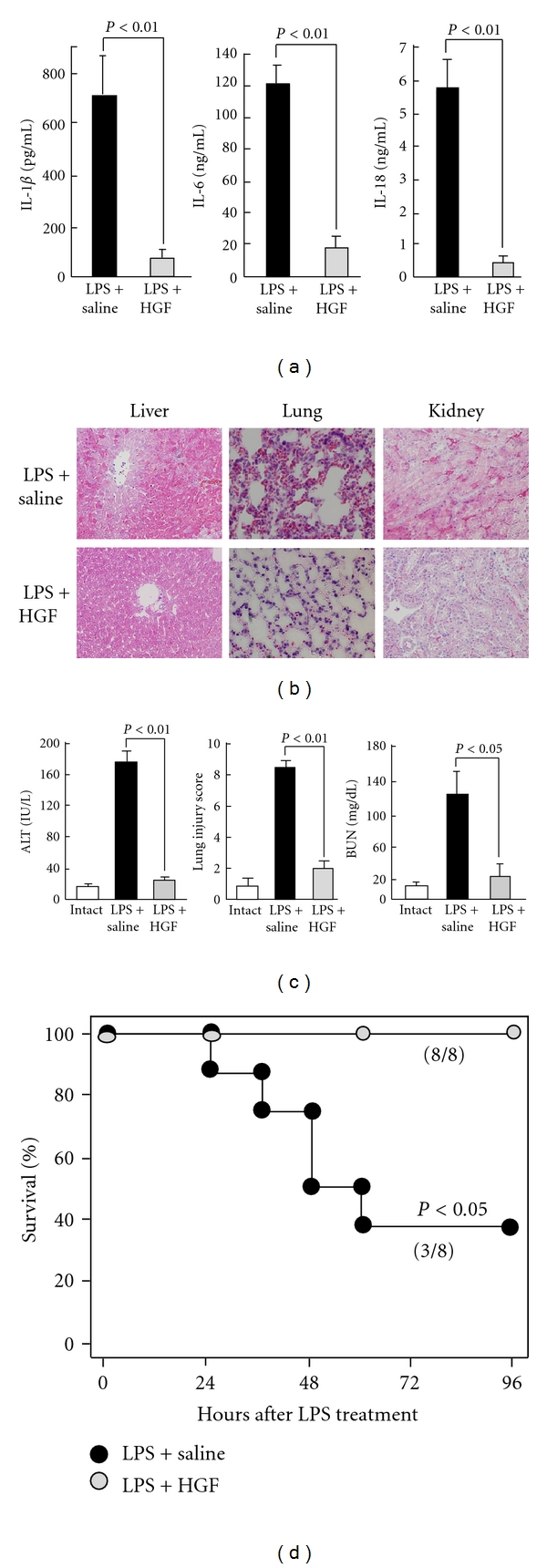
Therapeutic effects of recombinant HGF on MOF induced by LPS in mice. (a) Inhibitory effect of HGF treatment on cytokine storm. LPS-induced increases in plasma IL-1*β*, IL-6, and IL-18 levels were inhibited by HGF treatment. There are significant differences between saline- and HGF-treated septic groups (24 hours after challenge, ANOVA test). (b) Attenuations of histological lesions, such as hepatic degeneration, lung edema, and renal congestion by HGF in LPS-treated mice. (c) Prevention of hepatic, pulmonary and renal dysfunctions by HGF in septic mice. ALT: alanine aminotransferase. BUN: blood urea nitrogen. There are significant differences between saline- and HGF-treated septic groups (24 hours after challenge, ANOVA test). (d) Improved survival rate within 4 days after HGF treatment (*n* = 8 per group), with a significant difference (96 hours, Kaplan-Meier test). Overall, recombinant HGF was shown to prohibit septic MOF in a mouse model of sepsis [28, 29].

The lung is one of the most frequently identified organs to fail in sepsis and is also the most frequent primary site of infection [4]. The development of ARDS is common in those cases. ARDS is caused by protein-rich pulmonary edema that elicits severe hypoxemia. The lung injury is caused by primarily neutrophil- and platelet-dependent damage to the endothelial and epithelial barriers of the lung under pathological situations, including sepsis. LPS-treated rodents are widely used as an animal model of septic ARDS. Using this characterized model, we provide evidence that early administration of HGF may be useful for inhibition of septic ARDS [29]. In the saline-treated control group, neutrophil infiltration, edematous lesion and fibrin clotting were observed in the lung post-LPS challenge. In contrast, recombinant HGF strongly suppressed the onset of ARDS-like histological lesions seen in the LPS-treated mice. Within 24 hours post-LPS challenge, control mice suffered from the respiratory dysfunction with systemic cyanosis, resulting in a 40%-survival within 3 days. Of note, HGF administration blocked these clinical abnormalities [29]. As a result, more than 80% of septic mice were rescued by recombinant HGF treatments (Figure 2). Thus, HGF was shown to inhibit septic ARDS in mice via both anti-inflammatory and anti-coagulant pathways.

### 4.3. Inhibitory Effects of HGF Administrations on Septic ARF

ARF secondary to sepsis is a highly prevalent diagnosis in the ICU setting and continues to be associated with a high rate of morbidity (70%) [3]. The pathophysiology of septic ARF involves ischemic or toxic injury to the renal tubular epithelia, resulting in necrosis or apoptosis, and clinically is characterized as acute tubular necrosis. LPS-treated mice mimic the renal damage of human patients during sepsis. We first demonstrated that recombinant HGF strongly inhibited the onset of ARF in mice post-LPS challenge [29]. Actually, administration of recombinant HGF prohibited the LPS-mediated renal damages (such as neutrophil infiltration, tubular damage, and glomerular fibrin deposition), especially within 24 hours post-LPS challenge [29]. Consistent with the histological findings, HGF was shown to improve the renal dysfunction of LPS-challenged mice. This experimental result indicates that HGF supplement therapy might be available for inhibiting not only ARDS but also septic ARF, an important lethal factor of sepsis-induced MOF [3, 4].

### 4.4. Suppression of MOF by HGF in a Model of Polybacterial Sepsis

In the clinical bedsides of ICU, MOF occurs through a polybacterial infection. Thus, it is important to reevaluate the effect of HGF under pathological situations that mimic the clinical setting. Polybacterial MOF is inducible in animals by cecal ligation and puncture (CLP). Using CLP-treated rats as a model of septic peritonitis, Kondo et al. demonstrated the therapeutic efficacy of recombinant HGF, especially for attenuation of MOF. After the surgery of CPL, hepatic degeneration became evident in rats, along with leukocyte infiltration [45]. In contrast, recombinant HGF inhibited CLP-associated hepatitis in rats. In this process, HGF prevented the sepsis-induced loss of thrombomodulin, a key endothelial molecule that counteracts DIC formation. As a result, HGF supplement therapy improved the survival rate of rats under persistent peritonitis with poly-bacterial infections [45]. There is now ample evidence to show the protective effects of HGF during experimental sepsis in animal models (Table 3).

### 4.5. Safety Evaluations of HGF in Experimental Animals

It is important to discuss the possible side effects of HGF during septic conditions. Given that HGF produces anti-thrombotic effects via the upregulation of uPA [38], side effects, such as hemorrhage, may be alarmed. However, recombinant HGF improved the LPS-induced prolongation of prothrombin time in mice, with no signs of hemorrhage. Indeed, HGF did not shorten the prothrombin time below normal levels, as reported in the cirrhotic rodents [36]. Administrations of recombinant HGF into normal rats for 4 weeks did not modify the basal coagulation activity (data not shown), suggesting little effect of HGF on hemorrhage induction, but careful attentions should be paid to this issue for future clinical studies of HGF.

We further discuss the consequence of HGF-mediated suppression of inflammation during systemic infections. Of note, HGF prevents organ injuries in rats with systemic infection [45]. In the same CLP-treated model, HGF decreased the number of bacteria in blood, and as a result, SIRS was attenuated (unpublished data), implying other mechanisms of HGF for the possible enhancement of bacterial clearance, or for bacterial growth arrest. Thus, careful evaluation must be done to clarify the possible inhibitory effect of HGF on bacteremia in septic patients.

Whether HGF is procarcinogenic or not should also be discussed. Toxicological studies revealed that a daily intravenous injection of recombinant HGF for 4 weeks (i.e., 28 times) did not elicit tumor formation in rats or monkeys (unpublished data). Given that a duration period of intravenous HGF treatment during acute infection is probably within 7 days, we predict that the short-term injection of HGF will not increase a cancer risk, and clinical trials (Phase I) are now in progress with a careful focus on this issue. On the other hand, tumor stroma-derived HGF plays a central role in the invasion of cancer cells in growing tumors [10]. Thus, clinical use of HGF is contraindicated for patients who have already manifested growing tumors.

Upregulation of endogenous HGF is required for minimizing tissue injuries at least in rodents [10], while blood levels of HGF are increased in patients [16, 47, 48], thus suggesting a possible contribution of HGF upregulation to reducing septic damages. However, production levels of HGF seem insufficient in septic rats, with a time lag between the injury and HGF upregulation [19, 63]. Thus, HGF may be a candidate for the therapeutic development of septic diseases (Table 3), and large clinical studies are necessary to understand the regulation of HGF in humans before proposing HGF as a new drug target in such a complex system as sepsis.

## 5. Molecular Mechanisms of HGF-Induced Anti-Inflammation

TLR-mediated innate immune system triggers the onset of MOF during infectious diseases. In particular, cytokine storm, characterized by a dramatic increase in blood proinflammatory cytokines, is a fundamental event that provokes systemic coagulation and inflammation [67]. For example, TNF-*α* and IL-6 are known to be critical for inducing thrombosis in vascular vessels and necrosis/apoptosis in parenchymal epithelium. Chemokines, including IL-8, and adhesion molecules, such as ICAM-1, are also important for the rolling and extravasation of leukocytes between endothelial cells. These molecules are upregulated through a common transcriptional factor, NF-*κ*B [67]. We and other groups have revealed a novel function of HGF for suppressing NF-*κ*B activation in numerous types of cells [32, 41–43]. This section describes the molecular mechanisms whereby cytokine storm is attenuated by HGF, mainly focusing on c-Met-downstream pathways leading to inhibition of TLR4-mediated NF-*κ*B activation.

### 5.1. Prevention of SIRS by HGF through an HO-1 Pathway in Macrophages

Given that cytokine storm is responsible for coagulation and inflammation during sepsis, therapeutic effect of recombinant HGF may be due to the possible down-regulation of proinflammatory cytokines. Using LPS-treated mice, we found that c-Met levels increased in hepatic and pulmonary macrophages, a major contributor to cytokine storm [28, 29]. Of note, recombinant HGF targeted these macrophages at the early stage of SIRS and strongly blocked the LPS-mediated increases in blood IL-1*β*, IL-6, and IL-18 in septic mice [28, 29, 56].

Hemeoxygenase-1 (HO-1) is the rate-limiting enzyme for the oxidative degradation of heme into carbon monoxide, free iron, and biliverdin [68]. These products play pivotal roles in the attenuations of cytokine storm through NF-*κ*B inactivation, especially during sepsis [68]. In HO-1-deficient mice, blood IL-6 levels dramatically increased (>100-fold of wild-type) after the LPS injection [69], thereby identifying HO-1 as an essential suppressor of cytokine storm. We found that recombinant HGF enhanced LPS-mediated expression of HO-1 by the hepatic and pulmonary macrophages in septic mice, and was associated with decreases in blood IL-1*β*, IL-6, and IL-18 levels [28, 29]. Such an HGF-induced attenuation of cytokine storm was partially restored by an HO-1 inhibitory treatment [29]. As a result, LPS-mediated NF-*κ*B activation in resident and infiltrated macrophages was suppressed by HGF treatment.

**Figure 3:**
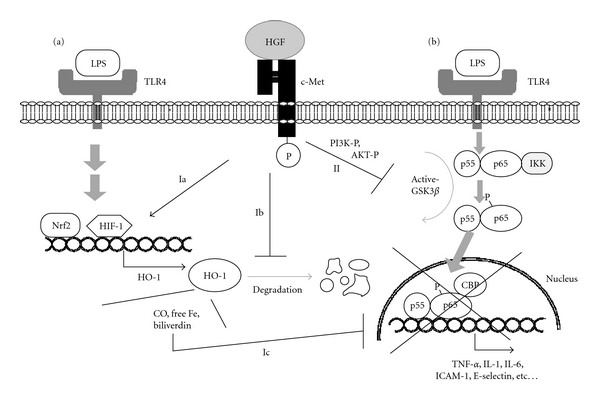
Molecular mechanisms of HGF-c-Met-mediated signaling for inhibiting TLR4-mediated NF-*κ*B activation in macrophages. (a) HO-1-dependent pathway. HO-1 is upregulated at a downstream of TLR4 through the activation of transcriptional factors, such as Nrf2 or HIF-1. During heme metabolism by HO-1, carbon monoxide (CO), free iron and biliverdin are generated, and these products attenuate NF-*κ*B activation (i.e., negative feedback system). In this process, c-Met signal enhances the HO-1 transcriptional pathway (1a) or stabilizes HO-1 from degradation (1b). Overall, HGF-mediated increases in HO-1 partially contribute to NF-*κ*B inactivation (1c). (b) GSK3*β*-inactivated pathway. LPS-TLR4-mediated signaling leads to nuclear localization and activation of NF-*κ*B via GSK3*β* activation pathway. In contrast, HGF-c-Met signaling elicits PI3K and AKT phosphorylation, and then GSK3*β* is inactivated. As a result, CBP, a coactivator of NF-*κ*B, is sequestered from the p65 subunit of NF-*κ*B, and transcription of inflammatory molecules, such as TNF-*α*, IL-6 and ICAM-1, is arrested.

Taken together, we predict that enhancement of HO-1 by HGF in macrophage is, in part, involved in the counteractive effect of HGF on TLR4-mediated inflammation. The increase in HO-1 by HGF in the presence of LPS is due to a posttranscriptional pathway (i.e., stabilization of HO-1), while HGF may increase HO-1 in septic organs via a transcriptional pathway. Thus, both pathways are important for HGF-mediated protection during sepsis (Figure 3(a)).

### 5.2. Counteractive Effect of HGF on TLR4-Mediated Inflammatory Signaling

We emphasized the involvement of HO-1 in HGF-mediated anti-inflammation, but its contribution is partial, especially *in vitro* [28], suggesting a direct inhibitory effect of HGF on the LPS-TLR4 signaling pathway. Activation of GSK3*β* is known to be required for promoting TLR4-mediated NF-*κ*B activation. Recently, Coudriet et al. provided evidence that inhibition of GSK3*β* activity by HGF leads to the inhibitory outcome of TLR4-mediated NF-*κ*B activation, as follows [46]. In a culture of bone marrow-derived macrophage, LPS caused a nuclear shift of NF-*κ*B (p55–p65 dimer), phosphorylation of p65 at serine-276, and then proinflammatory cytokines, such as IL-6, were over-produced. In this pathway, CREB-binding protein (CBP) is essential for NF-*κ*B activation as a coactivator that directly interacts with p65/NF-*κ*B [46]. In contrast, HGF elicited the phosphorylation of PI3K and AKT pathways at a c-Met downstream, and, more importantly, GSK3*β* activity was attenuated by HGF-mediated phospho-AKT. Consistently, NF-*κ*B activation (i.e., nuclear localization and p65 serine-276 phosphorylation) after LPS challenge was prohibited by HGF. In this process, c-Met signaling inhibited the binding of CBP to NF-*κ*B through enhancing a direct interaction between phospho-CREB and CBP. Under the HGF-mediated loss of NF-*κ*B-CBP complex, the transcription of IL-6 was arrested, even in the presence of LPS (Figure 3(b)). Such a direct effect of HGF on TLR-4 downstream is also important to explain the mechanisms whereby HGF inhibits cytokine storm.

### 5.3. Inhibition of Cytokine-Mediated NF-*κ*B Activation by HGF in Various Cells

We further discuss the suppressive effect of HGF on NF-*κ*B activation under pathological conditions. TNF-*α* is known to elicit NF-*κ*B activation, resulting in acceleration of MOF. When HGF is added into human endothelial cells in the presence of TNF-*α*, NF-*κ*B activation becomes faint in an HGF-dependent manner [32, 34]. As a result, inductions of ICAM-1 and E-selectin and leukocyte attachments are suppressed by HGF, contributing to an HGF-mediated improvement in kidney diseases in rodents [32, 33]. In this process, HGF rapidly activates nitric oxide production in cultured endothelial cells via inducing nitric oxide synthase (NOS), and this is associated with the decrease in E-selectin [34]. Notably, HGF-mediated NF-*κ*B inactivation and E-selectin downregulation are abolished by NOS-inhibitors [34]. Thus, the NO-dependent pathway may be involved in the counteractive effect of HGF on TNF-*α*-induced NF-*κ*B activation. HGF inhibits vascular endothelial growth factor- (VEGF-) induced NF-*κ*B activation [70]. These results indicate that HGF can attenuate further tissue inflammation and injury, even if cytokine storm occurs in the middle and late stages of septic diseases.

### 5.4. Other Mechanisms Involved in HGF-Induced Anti-Inflammation

We finally discuss other mechanisms of HGF-mediated anti-inflammatory effects. IL-10 is an anti-inflammatory cytokine: LPS-induced hepatitis is more evident in IL-10-knokout mice than in wild-type mice [71]. HGF promotes IL-10 production in LPS-stimulated macrophages via an HO-1-dependent pathway [28]. HGF-mediated enhancement of phospho-CREB-CBP interaction, as mentioned above, may participate in the IL-10 neo-induction [46]. Furthermore, HGF stimulates secretion of IL-1-receptor antagonist that can inhibit inflammatory events [72]. Monocyte chemoattractant protein-1 (MCP-1) is critical for recruiting macrophages, while HGF inhibits the MCP-1 expression *in vivo* [73] and *in vitro* [74]. These biological activities may also contribute to HGF-induced anti-inflammatory outcomes during sepsis.

## 6. Summary and Perspective

**Figure 4:**
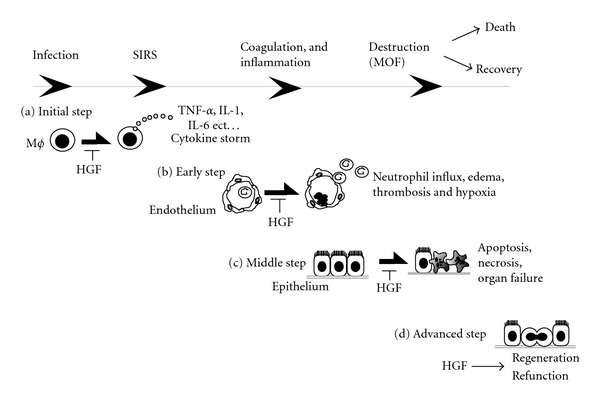
Step-by-step therapy using HGF in a chronology of septic phases including infection, SIRS, coagulation, and MOF. HGF inhibits the development of sepsis-induced MOF through different ways at different phases. (a) In an initial step, HGF targets endotoxin-primed macrophages and inhibits cytokine storm via indirect (i.e., HO-1 recruitment) and direct (i.e., GSK3*β* inactivation) pathways [29, 46]; (b) in an early step (i.e., post-cytokine storm), HGF protects endothelial cells from cytokine-induced inflammatory events, such as ICAM-1 and E-selectin overexpressions [32–34], and HGF also prevents DIC via the preservation of thrombomodulin on endothelial cells [44, 45]; (c) in a middle step (i.e., after coagulation), HGF protects resident functional cells from hypoxia or cytokines via early induction of Bcl-xL or Bcl-2 [40]; finally (d) in an advanced stage (i.e., after injury), HGF enhances tissue repair via enhancing mitogenesis and morphogenesis of surviving epithelial cells [10, 15].

This paper summarized new insights into HGF-mediated anti-inflammatory mechanisms during diseases, including sepsis. On the other hand, decoy oligonucleotides against NF-*κ*B are known to inhibit NF-*κ*B-dependent gene transcription by competing with *cis*-acting elements of inflammatory genes [75]. Several investigators have postulated the usefulness of “NF-*κ*B decoy” in various types of inflammatory diseases, including sepsis [76, 77]; however, its effect is limited to anti-inflammation. In addition to this effect [32, 41–43], HGF has numerous merits, such as anti-coagulation in endothelial cells, anti-apoptosis and regeneration in epithelial cells, and these effects may reduce the septic injury in each stage (Figure 4). Results obtained from animal studies suggest that HGF might be an interesting and therapeutic option.

Given that growth factors have various biological activities, growth factor therapy is still hopeful for treating sepsis; however, VEGF often elicits inflammation via activating NF-*κ*B pathways, as reported elsewhere [70]. Other growth factors, such as IGF-1 and HB-EGF, also enhance inflammation and apoptosis [78, 79]. HGF has beneficial effects opposite to these growth factors [32, 70]. Since the HGF activation system may be impaired during sepsis [80], intravenous injection of an active form of HGF (i.e., recombinant HGF) could be the primary choice for HGF supplemental therapy. Furthermore, transplantation of HGF-producing cells (e.g., HGF cDNA-transfected stromal cells [44]) may also be applicable at a sub-acute phase, although it will take several days for the cell preparation. Such a cell-specific HGF delivery system may reduce a hemodynamic effect of HGF, such as mild hypotension [81].

HGF may be present in a form of inactive precursor in blood [62]. Thus, HGF activators, such as uPA, may also be available for enhancing the effects of HGF. Supplementation of active HGF, as well as the enhancement of HGF activation, might be a new option for reducing MOF, and future clinical studies would shed more light on this hypothesis. Current clinical studies, focusing on the safety and effectiveness of HGF, have been tested in patients with various diseases worldwide [82–85]. HGF is now much more than a growth factor [86–88], and further basic and clinical studies using HGF will provide new insights into cell signaling networks connected to an attenuation of innate immune systems during inflammatory diseases, including sepsis. 

## Figures and Tables

**Table 1:** Biological effects of HGF on target cells, involved in sepsis-related MOF.

Target cells	Effect	Involved mechanism	References
*Epithelial cells*			
	Mitogenesis	Grab2-MAPK	[13]
	Migration	Gab1-PI3K	[13]
	Morphogen	Gab1-PI3K	[13]
	Anti-apoptosis	Bcl-xL induction	[15, 40]

*Vascular cells*			
Endothelium	Mitogenesis	MAP-kinase	[10]
	Anti-inflammation	Anti-NF-*κ*B	[32, 41–43]
	Anti-apoptosis	Bcl-2 upregulation	[10]
	Anti-coagulation	TM preservation	[44, 45]
Pericytes	Anti-proliferation	Anti-MAPK	[10, 15]

*Immune cells*			
Macrophages	Anti-cytokine storm	HO-1 upregulation	[28, 29]
	Anti-NF*κ*B activation	GSK3*β* de-phosphorylation	[46]
DC	Tolerogenic effects	TH1 ≪ TH2 balance	[10, 30]
Eosinophils	Anti-inflammation	Inhibition of eosinophilic toxin release	[10]
T-lymphocytes	Anti-proliferation	IFN-*γ* downregulation	[10]

Epithelial cells include hepatocytes, renal tubular cells, and alveolar and bronchial epithelium. TM: thrombomodulin; DC: dendritic cells; GSK3*β*: glycogen synthase kinase-3*β*. For abbreviations see text or original references.

**Table 2:** Increases in blood HGF levels in patients during various diseases.

Patients (P)	Healthy control (HC)	Ratio of P to HC (Fold increase)	Literature
*Sepsis*			
0.69 ± 0.47 ng/mL*	0.10 ± 0.03 ng/mL	6.90	Sakon et al. [47]
*SIRS (non-sepsis)*			
0.49 ± 0.37 ng/mL	0.10 ± 0.03 ng/mL	4.90	Sakon et al. [47]
*Septicemia*			
4.53–5.11 ng/mL**	0.7–0.77 ng/mL	approx. 6.5	Nayeri et al. [48]
*Skin infection*			
2.61–2.75 ng/mL	0.7–0.77 ng/mL	approx. 3.7	Nayeri et al. [48]
*Bacterial pneumonia*			
0.96 ± 0.27 ng/mL	0.29 ± 0.03 ng/mL	4.18	Maeda et al. [58]
*Acute hepatitis*			
0.45 ± 0.23 ng/mL	0.27 ± 0.08 ng/mL	1.67	Shiota et al. [59]
*Chronic hepatitis*			
0.40 ± 0.16 ng/mL	0.27 ± 0.08 ng/mL	1.48	Shiota et al. [59]
*Liver cirrhosis*			
1.05 ± 0.64 ng/mL	0.27 ± 0.08 ng/mL	3.89	Shiota et al. [59]
*Severe pancreatitis*			
2.30 ± 0.61 ng/mL	n.d.	n.d.	Ueda et al. [60]
*Ulcerative colitis*			
1.38 ± 0.11 ng/mL	n.d.	n.d.	Srivastava et al. [61]

*Average value ± SD, **Median-mean value, and n.d.: not done.

During organ injury, endogenous HGF is delivered through endocrine and paracrine pathways and contributes to an increase in blood HGF levels [10]. HGF seems to be present in blood in an inactive form (i.e., pro-HGF) [62]. Thus, a rapid administration of recombinant HGF (i.e., active HGF) may be recommended under pathological conditions. Pharmacological levels of recombinant HGF in blood can be sustained within a range of 3–30 ng/mL, by modifying the injection doses and their intervals [15, 32].

**Table 3:** Landmark studies to demonstrate the therapeutic effect of HGF on sepsis-related MOF in animal models.

Target disease	Animal model	Dose and routes	Outocomes	References
*Liver:*				
Septic fulminant hepatitis	LPS + GalN (mouse)	rh-HGF 3 mg/kg/d, ip	Inhibition of hepatic failure, anti-apoptosis, improved survival	Kosai et al. [40]
Septic fulminant hepatitis	LPS + GalN (rat)	Adeno-HGF, ip	Improved survival, anti-apoptosis, hepatoprotection	Nomi et al. [64]
Septic hepatitis	LPS (mouse)	rh-HGF 1 mg/kg, sc	Anti-apoptosis, HO-1 induction, IL-10 upregulation, IL-6 downregulation	Kamimoto et al. [28]
Septic hepatitis	LPS (rat)	HGF-producing cell implantation	Anti-coagulation, hepatoprotection	Seto et al. [44]
				Kaido et al. [65]
Septic hepatitis	LPS (rat)	rh-HGF 1 mg/kg, iv	Hepatocyte replication	Gao et al. [66]
Septic hepatitis	CPL (rat)	rh-HGF 1 mg/kg, iv	Improved survival, anti-coagulation, hepatoprotection	Kondo et al. [45]

*Kidney:*				
Septic ARF	LPS (mouse)	rh-HGF 1 mg/kg, sc	Improved survival, inhibition of ARF, reduced tubular injury, HO-1 induction	Kamimoto et al. [29]
Albuminuria	LPS (mouse)	rh-HGF 1 mg/kg, sc	Anti-inflammation, reduced albuminuria, nephrin preservation, podocyte protection	Kato et al. [56]

*Lung:*				
Septic ARDS	LPS (mouse)	rh-HGF 1 mg/kg, sc	Suppressed edema, reduced neutrophils, alveoloprotection, HO-1 induction	Kamimoto et al. [29]
Acute lung injury	LPS (mouse)	rh-HGF 1 mg/kg, iv	Enhancement of alveolar cell replication	Gao et al. [66]

GalN: D-galactosamine; rh-HGF: recombinant human HGF; Adeno-HGF: adenovirus vector carrying HGF cDNA; CPL: cecal ligation and puncture; sc, subcutaneous injection; iv: intravenous injection; ip: intraperitoneal injection. For other abbreviations, see text.
